# A molecular switch modulates assembly and host factor binding of the HIV-1 capsid

**DOI:** 10.1038/s41594-022-00913-5

**Published:** 2023-02-09

**Authors:** Randall T. Schirra, Nayara F. B. dos Santos, Kaneil K. Zadrozny, Iga Kucharska, Barbie K. Ganser-Pornillos, Owen Pornillos

**Affiliations:** 1 Department of Molecular Physiology and Biological Physics, University of Virginia, Charlottesville, VA USA; 2 Present Address: The Peter Gilgan Centre for Research and Learning, Hospital for Sick Children, Toronto, Ontario Canada

**Keywords:** Virology, Cryoelectron microscopy, Supramolecular assembly, Structure determination

## Abstract

The HIV-1 capsid is a fullerene cone made of quasi-equivalent hexamers and pentamers of the viral CA protein. Typically, quasi-equivalent assembly of viral capsid subunits is controlled by a molecular switch. Here, we identify a Thr-Val-Gly-Gly motif that modulates CA hexamer/pentamer switching by folding into a 3_10_ helix in the pentamer and random coil in the hexamer. Manipulating the coil/helix configuration of the motif allowed us to control pentamer and hexamer formation in a predictable manner, thus proving its function as a molecular switch. Importantly, the switch also remodels the common binding site for host factors that are critical for viral replication and the new ultra-potent HIV-1 inhibitor lenacapavir. This study reveals that a critical assembly element also modulates the post-assembly and viral replication functions of the HIV-1 capsid and provides new insights on capsid function and inhibition.

## Abstract (web summary)

The authors use single-particle cryo-EM to analyze the fullerene cone structure of the HIV-1 capsid. They identify a hexamer/pentamer switch that allows for cone assembly and modulates the ligand-binding properties of the capsid.

## Main

Upon entry into a new host cell, the HIV-1 capsid performs several essential functions, including shielding the genome from innate immune sensors^1^, promoting reverse transcription^2^ and transporting the core from the entry site at the plasma membrane to the integration site inside the nucleus^3,4^. The fullerene cone architecture of the HIV-1 capsid represents an extreme case of quasi-equivalence, in which essentially each of the 1,200 or more copies of the assembled viral CA protein occupies a different chemical environment and thus adopts a different structural configuration^5–7^. Along the body of the cone, the CA subunits are assembled on a hexagonal lattice, with the CA amino-terminal domain (NTD) making hexameric rings that are linked together by the carboxy-terminal domain (CTD). In this part of the capsid, conformational variability afforded by CA’s two-domain organization accounts for quasi-equivalence, which manifests in the continuously changing curvature of the hexagonal lattice along the body of the cone^7,8^. Twelve CA pentamers occupy sharp points of curvature—termed declinations—exactly 12 of which are required for the capsid to completely close^5^. The molecular rules that dictate CA hexamer or pentamer formation have not been previously elucidated. In other quasi-equivalent viruses that have been studied, the capacity of genetically identical subunits to form hexamers and pentamers is conferred by molecular switches^9,10^. Such a switch has not been previously found in retroviral CA proteins. Furthermore, the relative contributions of CA hexamers and pentamers to HIV-1 capsid interactions with host factors in the post-entry pathway are also unknown. To address these key gaps, we performed cryogenic electron microscopy (cryo-EM) and biochemical analyses of pentamer-containing in vitro capsids and their complexes with ligands that modulate HIV-1 capsid assembly, stability and function.

## Results

### Assembly and structural analysis of in vitro HIV-1 capsids

**Extended Data Fig. 1:**
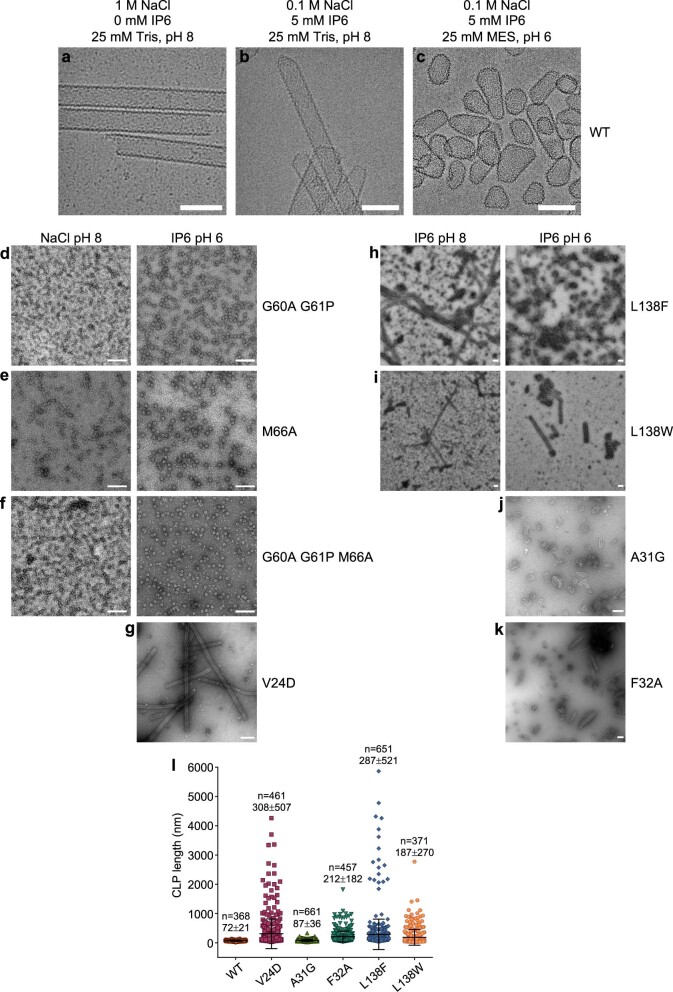
In vitro HIV-1 CA assemblies. (**a-c**) Cryoimages WT HIV-1 CA CLPs assembled under the indicated conditions, representative of at least two independent experiments with different protein preparations. Reactions were incubated at 37 °C for 1 h. Scale bars, 100 nm. (**d-k**) Negative stain images of CLPs formed under the indicated conditions, representative of 2 to 3 independent experiments with different protein preparations. Scale bars, 200 nm. (**l**) The longest dimension of individual CLPs were quantified for each of the indicated CA assemblies in 5 mM IP6, pH 6. According to one study, the average length of native fullerene cones is around 109 nm [ref. ^57^]. Aggregate data from all collected images are presented as the mean ± SD of the indicated n measurements. Source data

The classic in vitro model system for the HIV-1 capsid is tubular capsid-like particles (CLPs), which assemble when purified HIV-1 CA protein is incubated in high salt concentrations (≥1 M NaCl) and basic pH^6,11^. These tubes are made only of CA hexamers. Recently, the cellular metabolite inositol hexakisphosphate (IP6) has been shown to stabilize the HIV-1 capsid, by binding to the central channel of the CA hexamer^12,13^. IP6 also induces assembly of CA in vitro, even at physiological salt conditions^12^. We found that IP6-induced CLPs have a range of morphologies—capped tubes and cones—when assembled at pH 8, but are primarily conical at pH 6 (Extended Data Fig. 1). These observations indicate that IP6 promotes incorporation of CA pentamers into the assembling lattice, allowing formation of declinations that close the capsid shell. Low pH, in itself, does not efficiently induce CA pentamer formation in vitro^11,14^, but rather potentiates IP6’s ability to form these pentamers.

**Fig. 1:**
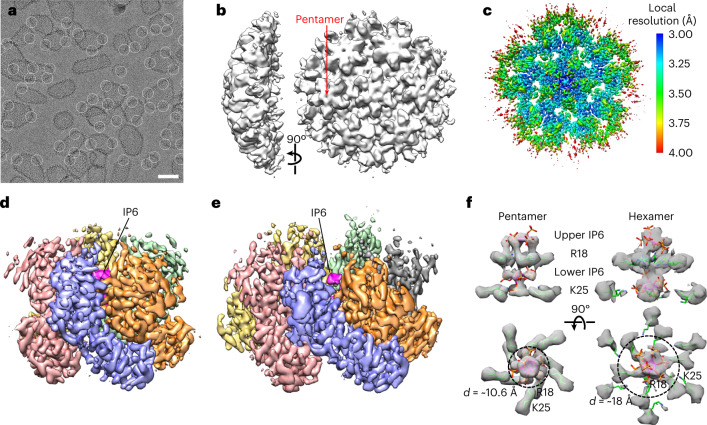
Assembly and structural analysis of IP6-induced HIV-1 CA CLPs. **a**, In vitro assembled CLPs, with declinations encircled. The image is representative of 14,940 micrographs. Scale bar, 50 nm. **b**, Initial ab initio map calculated from 670,480 particles. **c**, Map after further local refinement with imposed fivefold symmetry, colored according to local resolution. At this contour level (0.4), the 5 central pentamer subunits and their 15 closest hexameric subunits are very well defined. **d**, Structure of the pentamer (0.28 contour). Each subunit is in a different color, and IP6 is colored in magenta. **e**, Structure of the hexamer (0.22 contour). **f**, Side views (upper) and bottom views (lower) of the central channels of the capsomers. Maps (gray) are contoured at 0.4 (pentamer) and 0.21 (hexamer). Green sticks indicate the Arg18 and Lys25 side chains. IP6 molecules were rigid-body docked as the myo isoform. Dashed circles with indicated diameters (*d*) circumscribe the positions of the Lys25 Nε atoms.

**Extended Data Fig. 2:**
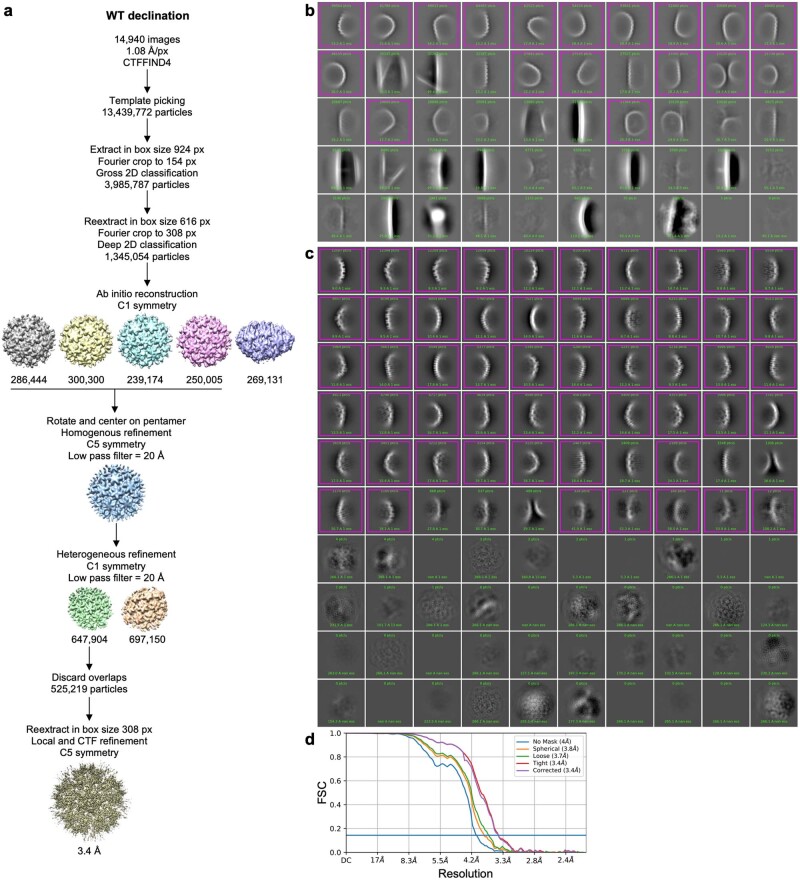
Single particle averaging workflow for the WT declination. (**a**) After the template picking step, particles were randomly split into 4 subsets to facilitate computations up to the ab initio reconstruction step. (**b**) Representative result of initial 2D classification of particles extracted in box size 924 px (998 Å). Magenta indicates classes selected for further processing. (**c**) Representative result of 2D classification of particles reextracted in box size 616 px (665 Å). (**d**) Fourier shell correlation (FSC) plot from the final refinement round. Source data

**Extended Data Fig. 3:**
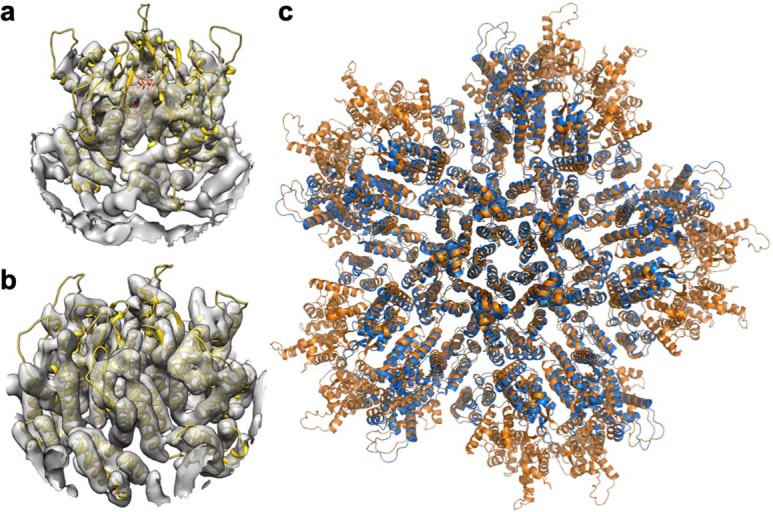
Structures of the capsomers in IP6-induced in vitro CLPs are similar to their corresponding structures in situ. (**a**) Docking of our real-space-refined CA pentamer model (yellow ribbons, with IP6 in sticks) into EMD-3466 (gray), the first reported in situ structure of the HIV-1 CA pentamer at 8.8 Å resolution^7^. (**b**) Docking of our real-space-refined CA hexamer model (yellow ribbons) into EMD-3465 (gray), an in situ structure of the HIV-1 CA hexamer at 6.8 Å resolution^7^. (**c**) Superposition of the final PDB model of the declination after real space refinement into our 3.4 Å map (orange) with PDB 5mcy (ref. ^7^) (blue).

By adapting deep two-dimensional (2D) classification particle selection^15^ and lattice-mediated alignment strategies^16^, we determined the structure of the declination from projection images of the IP6-induced conical CLPs (Fig. 1a,b, Extended Data Fig. 2, and Table 1). Local refinement with imposed fivefold symmetry resulted in a high-quality map at a nominal resolution of 3.4 Å, in which the central pentamer and surrounding hexamers are well-defined (Fig. 1c, Extended Data Fig. 2d, and Supplementary Video 1). Both the pentamer (Fig. 1d) and hexamer (Fig. 1e) are similar to their corresponding lower-resolution in situ structures that were determined by sub-tomogram averaging of capsids in intact virions^7,17^ (Extended Data Fig. 3). This indicates that the conical IP6-induced HIV-1 CA assemblies more closely mimic the architecture of the HIV-1 capsid than do previously described in vitro model systems.

**Table 1:** Data collection, processing and refinement statistics

	WT declination		WT declination + CPSF6-FG	T = 1 (CA-G60A G61P)	T = 1 (CA-M66A)	T = 1 (CA-G60A G61P M66A)
EMDB	EMD-26715		EMD-28186	EMD-28054	EMD-28057	EMD-26718
PDB	7URN		8EJL	8EEP	8EET	7URT
**Data collection, processing and map calculation**						
Magnification	×81,000		×81,000	×81,000	×92,000	×81,000
Voltage (kV)	300		300	300	200	300
Electron exposure (e^–^/Å^2^)	50		54	50	48	50
Defocus range (μm)	0.5 to 2.5		0.75 to 2.5	0.5 to 2.5	0.5 to 2.5	0.5 to 2.5
Pixel size (Å)	1.08		1.08	1.08	0.75	1.08
Symmetry imposed	*C*_5_		*C*_5_	*I*	*I*	*I*
Particles	525,219		166,463	494,057	116,627	509,666
Map resolution (Å)	3.4		3.9	2.2	3.1	2.4
FSC threshold	0.143		0.143	0.143	0.143	0.143
Map resolution range (Å)	2.3–8.3		2.5–8.9	2.2–4.9	2.1–7.2	2.4–5.2
**Coordinate modeling and refinement**						
	Chain A (pentamer)	Chains L,M,N (half-hexamer)				
Initial model	PDB 4XFX	PDB 4XFX	PDB 7URN	PDB 7URT	PDB 7URT	PDB 4XFX
Model resolution (Å)	3.4	3.6	4.1	2.5	3.4	2.5
FSC threshold	0.5	0.5	0.5	0.5	0.5	0.5
Map sharpening B-factor (Å^2^)	146.9	146.9	159.3	97.0	144.4	109.7
Model composition						
Non-hydrogen atoms	1,725	5,175	4,431	1,726	1,726	1,726
Protein residues	221	663	619	221	221	221
IP6	2	0	0	2	2	2
B-factors (Å^2^)	73.2	78.8	117.0	42.9	42.6	
Root mean squared deviations						
Bond lengths (Å)	0.006	0.004	0.005	0.002	0.004	0.004
Bond angles (°)	0.623	0.508	0.545	0.647	1.019	0.554
Validation						
MolProbity score	1.57	1.42	1.81	1.33	1.37	1.34
Clash score	8.40	5.79	7.23	3.95	6.23	4.05
Poor rotamers (%)	0.43	1.24	3.04	1.59	1.07	1.60
Ramachandran plot						
Favored (%)	97.26	97.87	97.82	100	99.54	99.09
Outliers (%)	0	0	0	0	0	0
*Z*-score	1.00	1.79	1.74	1.98	1.87	1.22

### IP6 stabilizes the pentameric HIV-1 CA ring

Both the hexamer and pentamer contain two IP6 molecules inside their central channels, one above and one below the ring of positively charged Arg18 side chains; the lower IP6 molecules are also coordinated by a second ring of Lys25 side chains (Fig. 1f). These support the model that charge neutralization promotes formation of the hexamer and pentamer. However, removal of the charges has different effects on CA assembly in vitro. Whereas the R18A substitution abolishes IP6-dependent assembly of CA altogether^12^, the K25A mutant is able to assemble tubes^18^. These observations suggest that Arg18 interactions stabilize both the hexamer and pentamer, whereas Lys25 interactions are more important for the pentamer. Consistent with this interpretation, the pentamer channel is narrower at the position of the Lys25 ring (dashed circles in Fig. 1f), allowing close, direct contacts between the primary amines of the lysine side chains and the phosphates of the lower IP6 molecule. Thus, our structure indicates that IP6 contacts with Arg18 also directly stabilize the CA hexamer, and contacts with both Arg18 and Lys25 stabilize the pentamer.

### Quasi-equivalence of the HIV-1 CA hexamer and pentamer

**Fig. 2:**
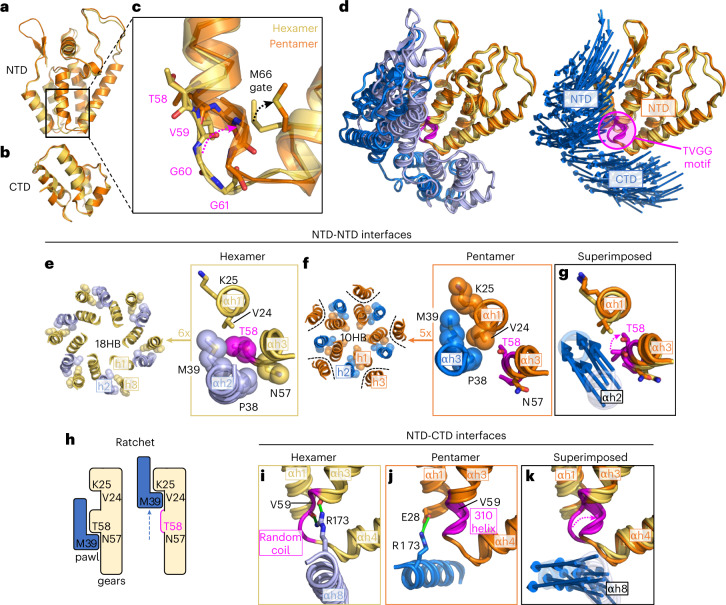
Comparison of HIV-1 CA pentamer and hexamer structures identifies a molecular switch. **a**,**b**, The NTD and CTD from hexameric (yellow) and pentameric (orange) subunits are superimposed. **c**, Distinct secondary structures of the TVGG motif in the hexamer and pentamer. **d**, The TVGG motif (magenta) is at the juncture of the NTD–NTD and NTD–CTD interfaces that hold together both capsomers. Reference pentamer and hexamer NTDs are superimposed. Right, displacement vectors (blue arrows) indicate positional changes of the NTD and CTD of the neighboring subunit. **e**–**g**, Details of the NTD–NTD interfaces in the hexamer (yellow and light blue) and pentamer (orange and blue). The dashed magenta arrow in **g** signifies overwinding of Thr58. Displacement vectors (blue arrows) signify ‘slippage’ of helix 2 from the hexamer state into the pentamer. 18HB, 18-helix bundle. **h**, Linear ratchet mechanism of hexamer/pentamer switching at the NTD–NTD interface. **i**–**k**, Details of the NTD–CTD interfaces in the hexamer (yellow and light blue) and pentamer (orange and blue). Green indicates hydrogen bonds. The dashed magenta arrow in **k** signifies refolding of the TVGG motif. Displacement vectors (blue arrows) indicate movement of helix 8. 10HB, 10-helix bundle.

**Extended Data Fig. 4:**
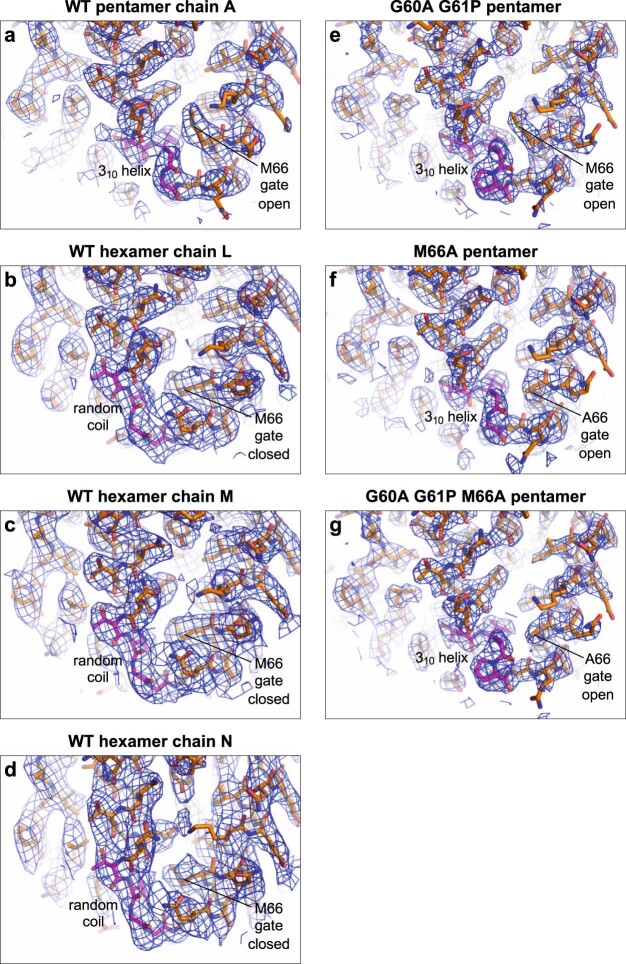
CryoEM maps (blue mesh) with real-space-refined models of asymmetric units (sticks). The TVGG motifs (and mutated versions) are in magenta. (**a**) WT pentamer. (**b-d**) WT hexamers. (**e**) G60A G61P pentamer. (**f**) M66A pentamer. (**g**) G60A G61P M66A pentamer.

In quasi-equivalent assembly systems, formation of different oligomers by the same protein is controlled by a conformational switch, which adopts different configurations that are evident from comparison of high-resolution structures of the quasi-equivalent states^19^. Superposition of the HIV-1 CA subunits in the hexamer and pentamer (Fig. 2a,b) allowed us to identify a putative switch in the NTD, comprising a Thr58-Val59-Gly60-Gly61 (TVGG) motif that adopts two alternative configurations—random coil in the hexamer, and 3_10_ helix in the pentamer (Fig. 2c). These configurations are well-defined in our cryo-EM map (Extended Data Fig. 4a–d and Supplementary Video 2). The interpretation that the TVGG motif functions as a switch is supported by its structural context: the motif mediates alternative packing of the NTD–NTD and NTD–CTD interfaces that hold together the capsomers (Fig. 2d,g,j).

In the hexamer, the NTD–NTD interface consists of a 3-helix repeating unit (helix 2 of one subunit and helices 1 and 3 of the adjacent subunit) that makes an 18-helix barrel (Fig. 2e). In the pentamer, helix 3 is excluded from the repeating unit, which then makes a ten-helix barrel (Fig. 2f). Refolding of the TVGG motif facilitates switching from the three-helix to the two-helix unit through a ratcheting mechanism. In the hexamer, Thr58 and Asn57 are the C-terminal residues of helix 3 and engage in reciprocal knobs-in-holes packing against Pro38 and Met39 in helix 2 of the neighboring subunit (Fig. 2e, boxed). In the pentamer, the protein backbone overwinds at Thr58 to facilitate the α-to-3_10_ helix transition (Fig. 2f, boxed). The conformational change disfavors the knobs-in-holes packing of helices 2 and 3, and instead Pro38-Met39 packs against Val24-Lys25 in helix 1. Thus, analogous to the ‘pawl’ of a ratchet, Pro38-Met39 in helix 2 engages two alternative ‘gears’: Asn57-Thr58 in helix 3 to form the hexameric NTD–NTD interface, or Val24-Lys25 in helix 1 to form the pentameric interface (Fig. 2g). Refolding of the TVGG motif toggles alternative packing by remodeling the hexamer gear, allowing the pawl to ‘slip’ into the pentamer state (Fig. 2h).

**Extended Data Fig. 5:**
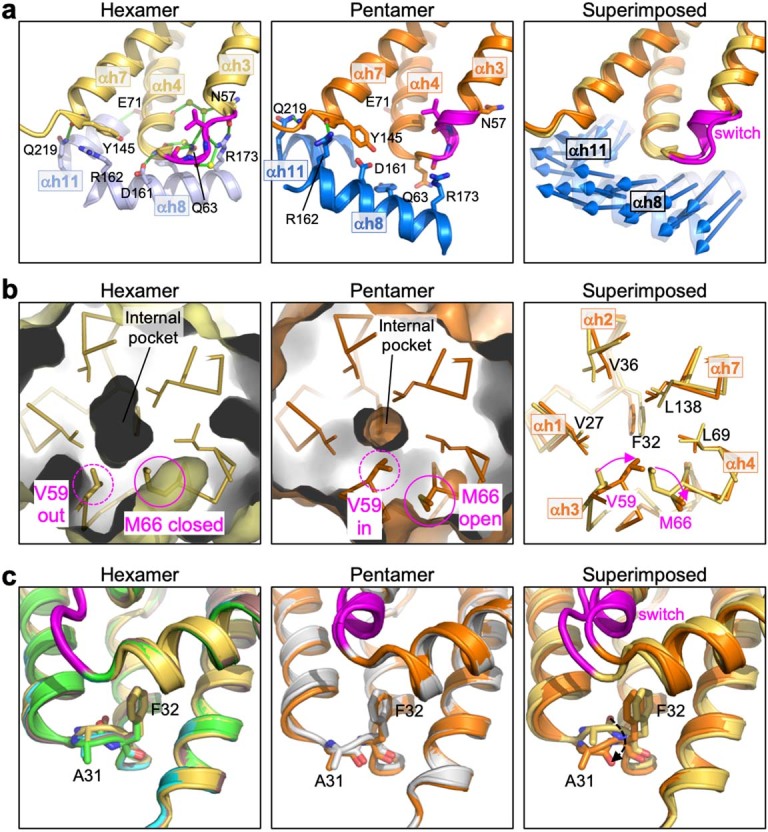
Additional distinguishing structural features of the HIV-1 CA hexamer and pentamer. (**a**) The NTD-CTD interfaces are distinguished by different sets of hydrogen bonds. Left: The hexameric interface is stabilized by an extensive network of hydrogen bonds involving the TVGG motif, surrounding residues and ordered water molecules. Shown is PDB 3h47, a high-resolution (1.9 Å) X-ray crystal structure^20^. Middle: In the pentamer, inter-subunit hydrogen bonds involving the TVGG motif are absent. Arg162 and Gln219 make hydrogen bonds with the C-terminal end of helix 7 and the linker, respectively. Right: Blue arrows indicate the displacement vectors for each residue in helices 8 and 11. (**b**) The NTD hydrophobic core is underpacked. The hexamer pocket (Left) is larger than the pentamer pocket (Middle). Right: Residues lining the pocket are shown as sticks and labeled. (**c**) Rotation of the Ala31-Phe32 peptide bond. Left: Hexamer structures, with PDB 3h47 in yellow. The three best-defined hexamer subunits in our WT CA declination structure are shown (chain L, green; chain M, cyan; chain N, brown). Middle: Pentamer structures of WT CA (orange, 3.4 Å) and G60A G61P M66A (gray, 2.4 Å). Right: PDB 3h47 is superimposed with the WT pentamer. Dashed arrow indicates rotation of the peptide bond.

The TVGG motif also mediates alternative packing of the NTD–CTD interface (Fig. 2i–k). In the hexamer, helix 8 of the CTD is adjacent to the helix 3–helix 4 loop (Fig. 2i). The highly conserved Arg173 residue in this helix makes two hydrogen bonds (green in Fig. 2i): one that caps the C-terminal end of helix 3 (Asn57) and another to the carbonyl of Val59. These are part of an extensive hydrogen-bonding network (Extended Data Fig. 5a) that facilitates pivoting of the CTD to accommodate the variable curvature of the hexagonal capsid lattice, as we have previously shown^20^. Our results suggest that the hydrogen-bonding network has an additional function in stabilizing both the random coil configuration of the switch and the α-helical character of helix 3. In the pentamer, the helix 3–helix 4 loop with the 3_10_ helix configuration no longer interacts with helix 8 owing to repositioning of the CTD (Fig. 2j). Instead, Arg173 makes hydrogen bonds (green in Fig. 2j) with a different partner (Glu28) in helix 1. Also, the C-terminal end of helix 7 (NTD) and portions of the NTD–CTD linker pack against the groove between helices 8 and 11 (CTD) (Extended Data Fig. 5a).

In summary, the TVGG motif within the helix 3–helix 4 loop of HIV-1 CA adopts two distinct secondary structural folds—random coil and 3_10_ helix—each of which engages a distinct set of quaternary packing interactions in the hexamer and pentamer. The location of the motif allows it to simultaneously modulate both the NTD–NTD and NTD–CTD interfaces (Fig. 2d,g,k), explaining how these two sets of interactions cooperate during capsid assembly.

### The TVGG motif remodels the hydrophobic core of the NTD

Refolding of the TVGG loop moves these four residues towards the protein hydrophobic core, and thereby changes or remodels the core. A large hidden pocket in the hexameric NTD core becomes smaller in the pentamer because the empty space is partly occupied by the repositioned Val59 side chain (Extended Data Fig. 5b). Proximal to the TVGG motif, Met66 changes its rotamer configuration to function as a gate (Fig. 2c). In the hexamer state, the Met66 gate is partly buried in the hydrophobic core in a ‘closed’ configuration and becomes exposed in an ‘open’ configuration to avoid clashing with the folded 3_10_ helix in the pentamer state. More distal to the TVGG motif, the Ala31-Phe32 peptide bond in the helix 1–helix 2 loop rotates by ~180° relative to the hexamer (Extended Data Fig. 5c). The Phe32 side chain points towards the hydrophobic core of the protein and lines the hidden pocket in both states. We propose that these conformational changes are functionally linked, and identify an allosteric network that communicates IP6-mediated interactions in helices 1 and 2 (NTD–NTD interface) to the TVGG switch.

### Importance of the TVGG motif and associated elements

All of the residues identified above are important for viral replication or inhibition. Substitutions for Val59 and Gly60 within the TVGG motif result in non-viable or severely impaired viruses^21–24^. Mutations in the Pro38-Met39 pawl in helix 2 abolish CA assembly^25^ or drastically lower the intrinsic stability of the capsid^26,27^. Asn57 and the Met66 gate modulate binding of the assembled capsid to host factors that are important for nuclear import and integration, as well as binding to one class of capsid-targeting inhibitors^28–37^. The hidden pocket in the NTD core that is walled by Phe32 and Val59 is also a binding site for a different class of inhibitors^21,38^. Thr58 and Met66 mutations and polymorphisms confer drug resistance, although at severe costs to viral fitness^21,34–36^. Thus, the TVGG motif is positioned at a critical site for capsid function and inhibition, indicating that it may have post-assembly functions as well.

### Designed manipulations of the CA hexamer/pentamer switch

**Fig. 3:**
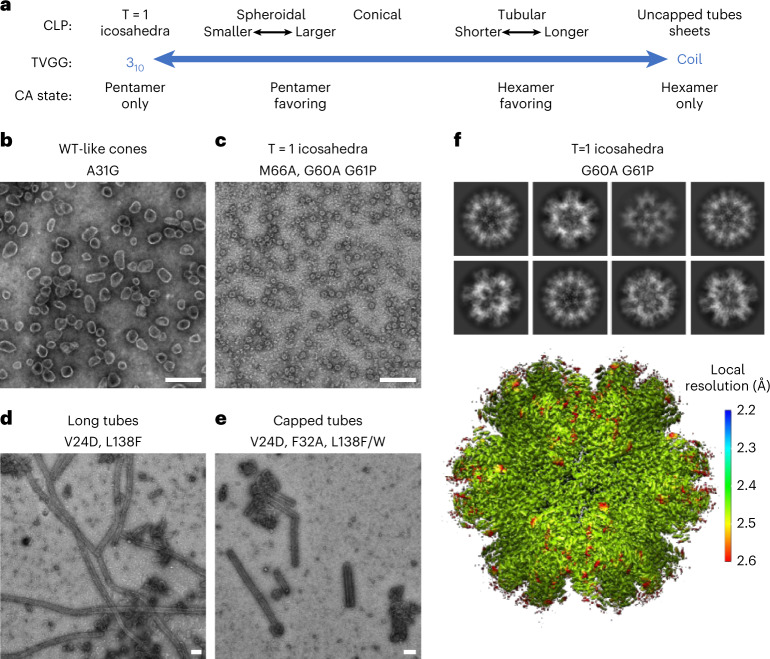
Designed manipulations that favor or disfavor pentamer formation during HIV-1 CA assembly. **a**, Predicted phenotypes of CA assembly behavior in vitro and CLP shape, as determined by the configuration of the TVGG motif. All but T = 1 icosahedra have been observed previously. **b**–**e**, Morphologies of CLPs formed by the indicated CA constructs in 25 mM MES, pH 6, 150 mM NaCl, 5 mM β-mercaptoethanol, and 5 mM IP6. Scale bars, 200 nm. **f**, 2D class averages (upper) and cryo-EM structure (lower) of T = 1 icosahedra made by CA-G60A G61P. The map is colored according to local resolution.

To test our hypothesis that the TVGG motif is a molecular switch, we designed structure-based mutations to control the coil/helix configuration, which should predict the in vitro assembly behavior of the CA protein and resulting CLP morphology (Fig. 3a). Solution conditions can also affect CLP morphology (Extended Data Fig. 1a–c), so for comparative analysis we used the IP6-induced conditions at pH 6, which produced conical CLPs with wild-type (WT) CA (Fig. 3b and Extended Data Fig. 1c).

**Extended Data Fig. 6:**
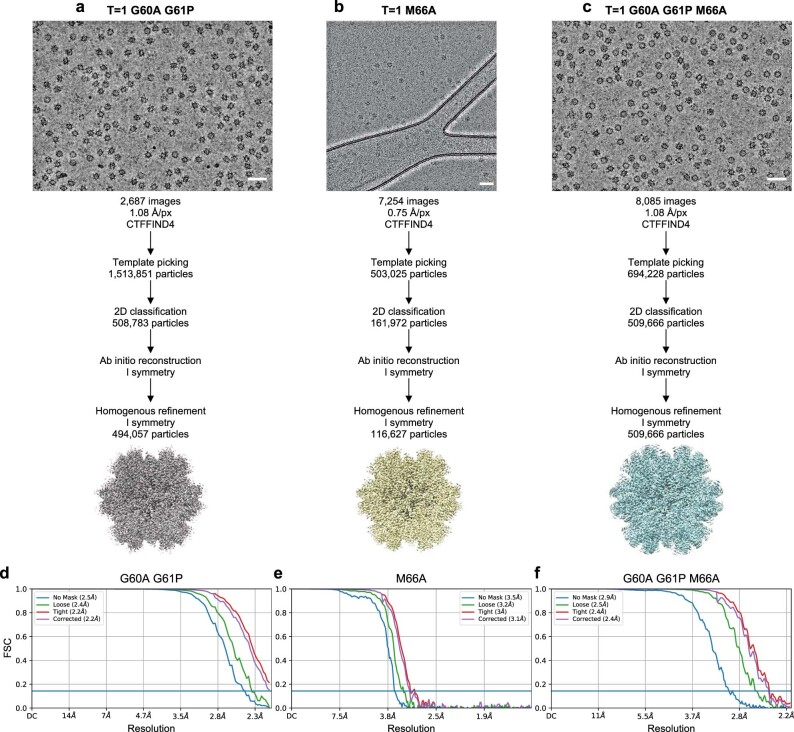
Single particle averaging workflow for T = 1 particles. (**a-c**) Workflows for indicated CA mutants. Scale bars, 50 nm. (**d-f**) Fourier shell correlation (FSC) plots from the final refinement rounds. Source data

WT HIV-1 CA is known to favor hexamer formation in vitro, implying that the 3_10_ helix configuration of the TVGG motif is normally disfavored. To stabilize the 3_10_ helix and thus favor the pentamer, we replaced the two glycines in the TVGG motif with alanine and proline. Crude modeling indicated that these two substitutions are compatible with the backbone configuration of the dipeptide in the pentamer state, but not the hexamer state. Indeed, incubation of CA-G60A G61P with IP6 induced assembly of small spheres of around 20 nm in diameter (Fig. 3c and Extended Data Fig. 1d). Cryo-EM imaging indicated that the spheres are T = 1 particles, or icosahedra made of 12 pentamers and no hexamers (Fig. 3f). Next, we surmised that the Met66 gate must constitute part of the activation barrier for coil-to-helix switching. Thus, a constitutively open gate (M66A) should also favor pentamers, and indeed the CA-M66A mutant also assembled into T = 1 particles (Extended Data Fig. 1e), as did the CA-G60A G61P M66A triple mutant (Extended Data Fig. 1f). We solved the cryo-EM structures of these T = 1 capsids to 2.2 Å (CA-G60A G61P), 3.1 Å (CA-M66A), and 2.4 Å (CA-G60A G61P M66A) (Fig. 3f, Extended Data Fig. 6 and Table 1). These confirmed that the mutant pentamers have the same structure as the WT pentamer, with the switch in the 3_10_ helix configuration and the gate in the open configuration (Extended Data Fig. 4e–g).

We then asked whether the CA mutants can still form hexamers by assembling them in 1 M NaCl and pH 8 conditions, in which WT CA assembles only open tubes (Extended Data Fig. 1a). CA-G60A G61P formed only aggregates, supporting the interpretation that the mutations locked the protein in its pentamer state (Extended Data Fig. 1d,f). Interestingly, the CA-M66A mutant also failed to assemble in three independent experiments (Extended Data Fig. 1e), although the mutation does not directly modify the TVGG switch. This result argues against a simple model wherein the TVGG motif fluctuates between the two states pre-assembly and then gets locked in upon assembly. We speculate that the random coil configuration may be analogous to a restrained spring that is poised to refold but is prevented from doing so by the closed Met66 gate.

We were unable to identify amino acid substitutions to rigidify the random coil configuration of the TVGG switch, which we predicted would have locked CA in its hexamer state. Nevertheless, structure-based mutations can be introduced to disfavor switching into the pentamer. As described above, Val24 stabilizes the pentameric NTD ring by mediating the knobs-in-holes interaction of helices 1 and 2 (Fig. 3d). Accordingly, the V24D mutation favored assembly of long, tubular CLPs when incubated with IP6 at pH 6 (Fig. 3d and Extended Data Fig. 1g,l).

Finally, we tested modifications to the protein hydrophobic core and its potential allosteric role in hexamer/pentamer switching. We predicted that increasing the packing density of the core would inhibit core remodeling and pentamer formation. To achieve this without modifying the TVGG motif, we replaced Leu138 with larger side chains; this residue is in helix 7, across the internal pocket from Val59, and does not directly interact with the switch (Extended Data Fig. 5b). The L138F, L138W and L138Y mutations significantly destabilized CA, yet the L138F and L138W mutants could still form cones. However, the CLPs were generally longer than the WT, indicative of impaired pentamer formation (Fig. 3d,e and Extended Data Fig. 1h–i,l). L138F also resulted in very long (>1 μm) tubes. Although there were fewer of these tubes, each contains tens of thousands of CA subunits in the hexamer state, again indicative of a lower propensity to form pentamers. We also tested the importance of the conformational change in the helix 1–helix 2 loop involving Ala31 and Phe32 (Extended Data Fig. 5c). As with the Leu138 substitutions, A31G and F32A also destabilized the protein and reduced assembly efficiency, yet still supported cone formation. The F32A mutation led to a higher proportion of tubular capsids, again indicating that pentamer formation is disfavored (Extended Data Fig. 1j–l). These results support the idea that the protein hydrophobic core allosterically connects helix 1, where IP6 binds, and the TVGG switch.

### The HIV-1 CA hexamer/pentamer switch modulates ligand binding

**Fig. 4:**
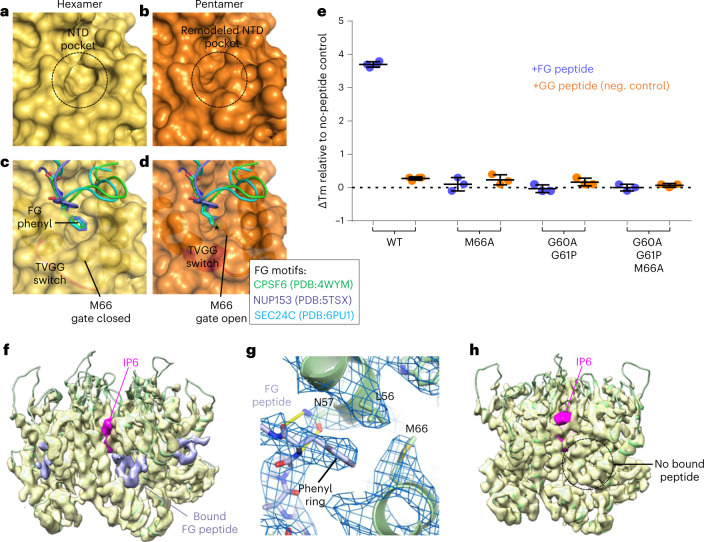
Conformational switching of the TVGG motif remodels the FG-motif binding site. **a**,**b**, Connolly surface representations of our WT hexamer and pentamer structures. **c**,**d**, The hexamer and pentamer are superimposed with the indicated crystal structures of CA with bound FG ligands. * indicates steric clash. **e**, Thermostabilization of indicated CLPs by CPSF6-FG and CPSF6-GG peptides, presented as mean ± s.d. of technical replicates (*n* = 3; *n* = 4 for WT). Data are representative of two biological replicates. Tm, apparent melting temperature. **f**,**h**, Views of the hexamer and pentamer from a cryo-EM reconstruction (3.9 Å) of the declination from WT CLPs incubated with excess CPSF6-FG peptide (0.4 contour). Pink indicates bound IP6, found in both the hexamer and pentamer. Lavender indicates that the peptide is bound only to the hexamer. **g**, Close-up of the binding interaction in the hexamer pocket. Source data

One of the critical functions of the HIV-1 capsid is to recruit host cell factors that promote viral replication in terminally differentiated T cells and macrophages, the natural hosts of HIV-1. Because these interactions are typically studied biochemically and structurally in context of the HIV-1 CA hexamer, and because genetic approaches cannot distinguish between the hexamer and pentamer, any potential contributions from the pentamer have been unknown. A major class of such capsid-binding factors includes CPSF6, NUP153 and SEC24C, each of which contains one or more phenylalanine-glycine (FG) motifs that bind within the NTD–CTD interface of the hexamer^29–31,33,37,39,40^. Binding of these natural FG ligands is competitively inhibited by the pharmacological inhibitors PF74 (refs. ^30–32,40^) and GS-CA1/GS-6207/lenacapavir^34–36^, each of which contains an analogous phenyl ring. The FG-motif phenylalanine ring binds to a hydrophobic pocket on the surface of the NTD that is adjacent to the helix 3–helix 4 loop containing the TVGG switch (Fig. 4a,c). This NTD pocket alone is sufficient for weak binding^28,29,40^. Tighter binding is afforded by additional contacts with the CTD, which adopts a closed configuration and contacts ligand moieties outside of the phenyl ring^30,31^. In essence, the NTD pocket makes the basal interaction with the FG motif or its equivalent (μM affinity), whereas surrounding elements contribute to tighter binding (pM for lenacapavir^35^).

**Extended Data Fig. 7:**
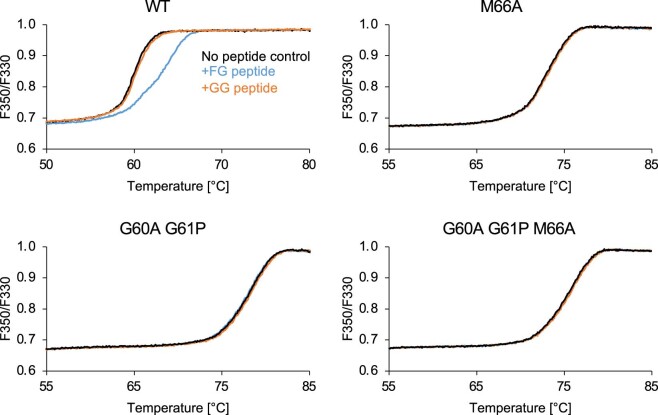
Representative raw thermal melting profiles. Indicated CLPs (12–22 μM) were heated in the absence of peptide (black curves), in the presence of 500 μM FG peptide (blue curves) or in the presence of 500 μM GG peptide (orange curves). The FG peptide induced a clear shift in the melting profile of WT CLPs (upper left panel), thus indicating binding. In all other cases, the melting curves are virtually superimposable with their respective no-peptide controls, indicating lack of binding. Source data

Our structures indicate that the alternative configurations of the TVGG motif remodel the NTD pocket (compare Fig. 4a and Fig. 4b). Furthermore, superpositions with published structures of CA in complex with FG-motif peptides indicate that the closed-gate configuration of Met66 in the hexamer is compatible with binding (Fig. 4c), but the open-gate configuration moves the Met66 side chain into the phenylalanine pocket, causing a steric clash with the ligand’s phenyl ring (Fig. 4d). Thus, the CA pentamer should be impaired in binding FG motifs. To assess whether pentamers and hexamers have different FG-binding properties, we measured the thermostability of the CLPs in the presence or absence of a representative FG-motif ligand, a peptide derived from the host factor CPSF6 (refs. ^29–31^) (Fig. 4e and Extended Data Fig. 7). To ensure that all signals come from assembled CA, CLPs were first purified from unassembled protein and aggregates by using size-exclusion chromatography. In accordance with our prediction, the CA-M66A, CA-G60A G61P and CA-G60A G61P M66A T = 1 particles were not stabilized by the FG peptide. In contrast, WT CLPs were stabilized by FG peptide, but not by a negative control GG peptide in which the FG phenylalanine was replaced by glycine. These results indicate that CLP binding to FG motifs requires the CA hexamer and that the CA-mutant pentamers lost binding, but do not rule out the formal possibility that WT CA pentamers can still bind FG.

**Extended Data Fig. 8:**
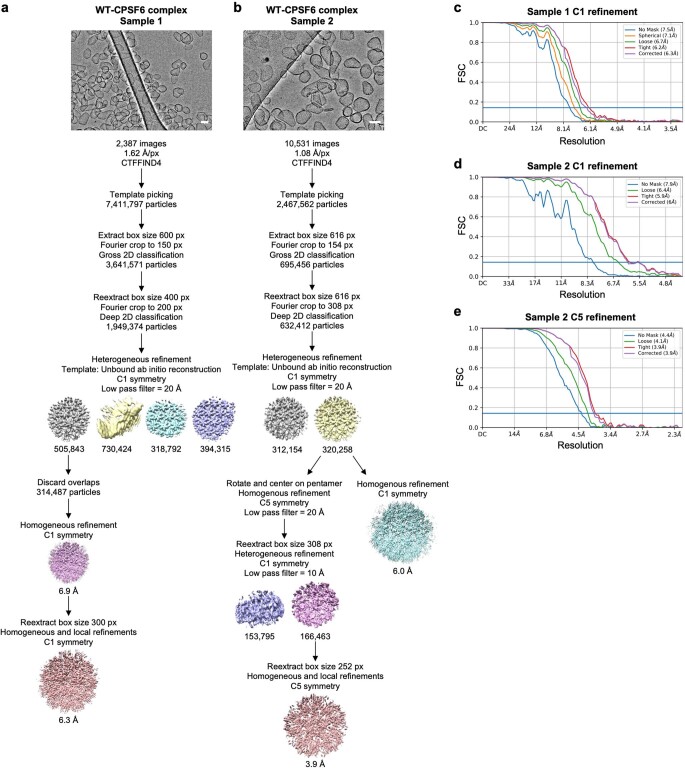
Single particle averaging workflow for the WT declinations in complex with CPSF6-FG peptide. **(a**, **b)** Workflows for indicated samples. Scale bars, 50 nm. (**c-e**) Fourier shell correlation (FSC) plots. Source data

**Extended Data Fig. 9:**
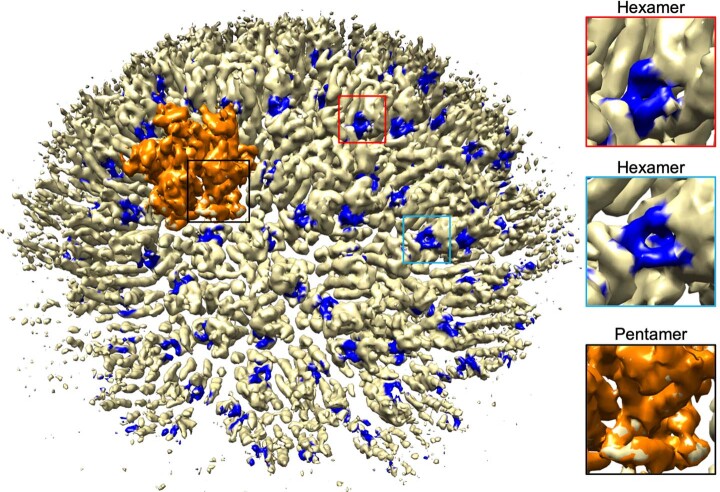
CryoEM assay of CPSF6-FG binding. The cryoEM structure (Sample 2, 6 Å resolution) of the WT declination in complex with CPSF6-FG peptide is shown at 0.4 contour. The pentamer is in orange. Peptide densities are in blue.

Because bulk biochemical assays cannot distinguish between pentamers and hexamers in WT CLPs, we assayed for differential binding using cryo-EM. We incubated the WT CLPs at high concentration (0.27 mM CA) with 11-fold excess FG peptide to ensure saturation of available binding sites. Images of the complexes were collected for two independent samples, and then structures (6.0- and 6.3-Å resolution) of the declinations were solved, imposing no symmetry and using a large box size that encompassed 1 pentamer and 17 hexamers (Extended Data Fig. 8a,b). The hexamers serve as an internal positive control, and indeed bound peptides were clearly seen as U-shaped densities within all of the resolved hexameric NTD–CTD interfaces (Extended Data Fig. 9). To visualize the detailed interactions, we refined one data set to 3.9-Å nominal resolution (Fig. 4f–h, Extended Data Fig. 8b and Table 1). The FG peptide bound in the expected configuration to the hexamers (Fig. 4f), with the FG phenylalanine and CA Met66 side chains clearly resolved by the map (Fig. 4g). We did not find peptide densities within the pentameric NTD–CTD interfaces (Fig. 4h and Extended Data Fig. 9). We therefore conclude that the CA pentamer does not bind to the CPSF6-FG motif and, by extension, is unlikely to bind other FG-containing host factors, such as NUP153 or SEC24C.

## Discussion

Here, we identify the conformational switch that modulates the ability of the HIV-1 CA protein to generate the hexameric and pentameric capsomers in the viral capsid. In other virus capsid proteins, amino and carboxyl termini are well-characterized switches that typically undergo order/disorder transitions^9,10,41,42^. The CA termini also function as order/disorder switches that are triggered by programmed proteolysis^43,44^, but these modulate conversion of the immature capsid into the mature capsid and not hexamer/pentamer switching. The TVGG motif in CA is distinct from these known switches because it is an internal loop that refolds from one ordered configuration to another.

Although the exact trigger of the TVGG switch within virions remains to be established, our in vitro assembly data indicate that IP6 can perform this function. This is in line with other viral capsid switches, which are also sensitive to the assembly environment^9^. IP6 binds CA helix 1 at the central channels of the hexamer and pentamer, and so must trigger the TVGG switch through allostery, again a common theme in virus capsid assembly^19,45^. In principle, allostery between IP6 and the TVGG switch can occur as the NTD–NTD and NTD–CTD interactions cooperatively form during assembly. Interestingly, however, our data indicate that allosteric communication may run through the protein hydrophobic core. Specifically, we find that: (1) refolding of the TVGG motif also remodels the core, (2) the helix 1–helix 2 loop changes conformation, (3) this loop includes Phe32, whose side chain is buried in the core, and (4) CA mutations that alter the core retain assembly but impair switching. These observations support the idea that the TVGG motif is allosterically connected to helix 1 by the hydrophobic core. A similar phenomenon has been documented in protein kinases, in which core residues act as a non-covalent bridge between two elements (called ‘splines’) located on opposite sides of the protein fold^46^. We speculate that CA’s helix 1–helix 2 (NTD–NTD interface) and helix 3–helix 4 (NTD–NTD and NTD–CTD interfaces) are analogous ‘splines’ bridged by the residues that line its loosely packed hydrophobic core. Allostery in kinases has been successfully exploited for pharmacological inhibition^47,48^, and similar strategies may also be applicable to HIV-1 CA.

Another important insight from these studies is our discovery that the HIV-1 CA hexamer/pentamer switch modulates not just the assembly process, but also the capacity of the assembled capsid to bind FG-motif host cell factors that are important for the post-entry pathway of the virus. Our results suggest that CPSF6 and other FG-motif host cell factors, which are implicated in HIV-1 nuclear import and integration site selection^49^, bind only to the hexamer. Other studies indicate that pentamer-specific factors might facilitate core trafficking in the cytoplasm or docking to the nucleus^3,50^, pointing to division of labor between the two capsomers. Taken together, these observations support the requirement for an intact or nearly intact capsid at these viral replication steps.

Lenacapavir, a new ultra-potent HIV-1 inhibitor, binds to the same site on the capsid as FG motifs. Indeed, a single point mutation in the methionine gate (M66I) confers resistance to this drug, although at severe cost to viral fitness^35,36^. The molecular basis of this fitness cost can now be explained in light of our findings.

## Methods

### Protein purification and assembly

HIV-1 CA proteins were purified as has been described^51^. Conical CLPs were assembled by incubating the protein (12–20 mg/mL) in 4–5 mM IP6, 50 mM MES, pH 6, 150 mM NaCl, 5 mM β-mercaptoethanol at 37 °C for 1–2 hours. Tubular CLPs were assembled by incubating the protein in 50 mM Tris, pH 8, 1 M NaCl, 5 mM β-mercaptoethanol at 37 °C for 1–2 hours. CA mutants were purified and assembled in the same way as the WT protein. Peptide-bound WT CLPs were prepared by diluting assembled CA (0.54 mM in 4 mM IP6) twofold into buffer containing 3 mM CPSF6-FG peptide (GTPVLFPGQPFGQPPLG, N-terminally acetylated and C-terminally amidated; Celltein), followed by incubation for 30 minutes on ice.

### Cryo-EM structure determination

Conical CLPs (with or without bound FG peptide) were diluted 8- to 12-fold with 25 mM MES, pH 6, 100 μM IP6 and then immediately applied (4 μL) to glow-discharged lacey carbon 300-mesh Cu grids. T = 1 particles were purified from unassembled protein using size-exclusion chromatography (Superdex 200 10/300 GL; Cytiva) in 25 mM MES, pH 6, 50 mM NaCl, 10 mM β-mercaptoethanol, 100 μM IP6, and 4 μL of relevant fractions were applied to C-flat 1.2/1.3 300-mesh holey carbon grids. Grids were briefly blotted manually and plunge-frozen in liquid ethane using a home-built device.

Cryo-EM data were collected at the University of Virginia Molecular Electron Microscopy Core. For the WT CLPs, CA-G60A G61P T = 1 particles, and CA-G60A G61P M66A particles, videos were collected using a Krios (ThermoFisher) operating at 300 kV and equipped with an energy filter and K3 direct detector (Gatan). Data were collected using EPU (ThermoFisher) at a pixel size of 1.08 Å in counting mode, with a total dose of 50 electrons/Å^2^ over 40 frames and target defocus of −0.5 to −2.5 μm. For CA-M66A T = 1 particles, videos were collected using a Glacios (ThermoFisher) operating at 200 kV and equipped with a Falcon 4 detector (ThermoFisher), at a pixel size of 1.5 Å in superresolution mode (effective pixel size of 0.75 Å), with a total dose of 48 electrons/Å^2^. For CPSF6-bound CLPs, we collected two data sets using the Krios on two independent samples: sample 1, at a pixel size of 1.62 Å and total dose of 54 electrons/Å^2^, and sample 2, at 1.08 Å and 50 electrons/Å^2^.

All image processing and map calculations were performed in cryoSPARC v.3.3.1–3 (ref. ^52^) (Table 1). Raw movies were corrected for beam-induced motion using MotionCor2 (ref. ^53^), and CTF estimation was performed with CTFFIND4 (ref. ^54^), as implemented in cryoSPARC. Initial particles were manually picked to generate references for subsequent template-based picking.

For the WT CA declination structure, 13,439,772 particles were initially extracted in box size 924 px (998 Å) (Extended Data Fig. 2a). Two rounds of reference-free 2D classification and selection were performed to identify and remove junk particles (mostly carbon edges) (Extended Data Fig. 2b). The selected 3,985,778 particles were then re-extracted in box size 616 px (665 Å) and processed through several rounds of iterative reference-free 2D classification and selection (Extended Data Fig. 2c). Parameters were adjusted with the goal of having well-defined classes, each with around 500–5,000 particles in the final round. This resulted in 1,345,054 particles that were used for ab initio structure calculation (5 models, *C*_1_ symmetry). Four models (1,075,923 particles) converged on similar maps and were combined into a single particle set through one round of refinement with *C*_1_ symmetry and a low-pass filter of 20 Å. The resulting map was then rotated and centered on the pentamer. Heterogeneous refinement (3D classification, 2 models) was then performed in *C*_1_, with a low-pass filter of 20 Å. After discarding overlaps, the final set of 525,219 particles was re-extracted in box size 308 px (333 Å) and refined with *C*_5_ symmetry and local CTF estimation, resulting in a map at a nominal resolution of 3.4 Å (Extended Data Fig. 2d).

For the T = 1 icosahedra, 503,025 (CA-M66A), 1,513,851 (CA-G60A G61P), and 694,288 (CA-G60A G61P M66A) particles were extracted (Extended Data Fig. 6). After two or three rounds of reference-free 2D classification, 116,627 (CA-M66A), 494,057 (CA-G60A G61P), and 509,666 (CA-G60A G61P M66A) particles were used for ab initio calculation with *I* symmetry imposed. Homogeneous refinement resulted in final maps at nominal resolutions of 3.1 Å (CA-M66A), 2.2 Å (CA-G60A G61P), and 2.4 Å (CA-G61A G61P M66A) (Extended Data Fig. 4e–g).

For the low-resolution FG peptide-bound structures, the particle sets after junk removal and 2D classification were processed through one round of heterogeneous refinement (using as reference two copies of a WT map filtered to 20 Å) (Extended Data Fig. 8). For sample 1, further alignment of 314,487 particles with *C*_1_ symmetry and box size 400 px (648 Å) produced a map at ~6.3-Å resolution (Extended Data Fig. 8c). For sample 2, alignment of 320,258 particles with *C*_1_ symmetry and box size 616 px (665 Å) produced a map at ~6.0-Å resolution (Extended Data Fig. 8d). Sample 2 was then reprocessed through another round of heterogeneous refinement to obtain a final set of 166,463 particles (box size 308 px, 333 Å), which was then refined with *C*_5_ symmetry to a nominal resolution of 3.9 Å (Extended Data Fig. 3e).

### Model building and refinement

Coordinate models were built for the WT declination by docking PDB 4XFX (ref. ^32^) into the maps, followed by iterative manual rebuilding using Coot^55^ and real-space refinement using Phenix^56^ (phenix.real_space_refine). Refinements were performed separately for two groups: the pentamer subunit (chain A) and the three closest hexamer subunits to the pentamer (chains L, M, and N) (Table 1). Non-crystallographic symmetry restraints were used when appropriate. The remaining hexamer subunits (chains O, P, and Q) were modeled by rigid-body docking of the final refined model of chain M as polyalanine.

Modeling of the WT declination in complex with FG peptide was performed by rigid body docking of chains A, L, M and N of the final refined WT model into the 3.9-Å map. The peptide was initially obtained by docking PDB 4WYM chain M (ref. ^31^) into the appropriate densities. Real-space refinement was performed as described above.

For the T = 1 particles, one copy of PDB 4XFX was docked into the CA-G60A G61P M66A map, followed by iterative manual rebuilding and real-space refinement. One round of morphing was performed in the first refinement iteration. To obtain CA-M66A and CA-G60A G61P models, the final refined model of the triple mutant was fitted through one round of rigid-body refinement. The mutated regions were rebuilt and refit manually, and then the models were subjected to two or three rounds of real-space refinement.

In all maps, modeling of IP6 densities was restricted to rigid-body fitting of the myo isoform, as described previously^12^.

Figures were made with Chimera or MacPyMOL (Delaglio Scientific).

### Negative-stain EM

Samples (4 μL) were applied to Formvar continuous carbon 300-mesh Cu grids and incubated for 2 minutes. Grids were moved to a 20-μL drop of 0.1 M KCl, incubated for 2 minutes and blotted. Grids were then placed on a 20-μL drop of 2% uranyl acetate, incubated for 2 minutes and blotted until they were dry. Images were collected on a Tecnai Spirit (ThermoFisher) or an F20 microscope (ThermoFisher), both operating at 120 kV.

### Nanodifferential scanning fluorimetry

Thermostabilization assays were performed using CLPs purified through size exclusion in 20 mM Tris, pH 8, 50 mM NaCl, 40 μM IP6. CLPs were diluted in the same buffer (to final μM concentrations as indicated) containing FG peptide (500 μM), GG peptide (500 μM) or no peptide. Samples were incubated for 5 minutes, then analyzed using a Tycho NT.6 (NanoTemper). Apparent melting temperatures were determined from raw curves using the manufacturer’s software.

### Reporting summary

Further information on research design is available in the Nature Portfolio Reporting Summary linked to this article.

## Online content

Any methods, additional references, Nature Portfolio reporting summaries, source data, extended data, supplementary information, acknowledgements, peer review information; details of author contributions and competing interests; and statements of data and code availability are available at 10.1038/s41594-022-00913-5.

## Supplementary information

Reporting Summary

Supplementary Video 1 Animation of the cryo-EM map of the WT declination with pentamer and hexamer models.

Supplementary Video 2 Comparison of the capsomers and identification of the TVGG switch.

## Data Availability

Cryo-EM maps have beeb deposited at the Electron Microscopy Data Bank (EMDB) under accession numbers EMD-26715 (WT declination), EMD-28054 (T = 1 CA-G60A G61P), EMD-28057 (T = 1 CA-M66A), EMD-26718 (T = 1 CA-G60A G61P M66A) and EMD-28186 (WT declination bound to CPSF6-FG peptide). Coordinates have been deposited at the Protein Data Bank (PDB) under accession numbers 7URN (WT declination), 8EJL (WT–FG complex), 8EEP (CA-G61A G60P), 8EET (CA-M66A) and 7URT (CA-G60A G61P M66A). The following datasets were used in this study: EMD-3465, EMD-3466, PDB 5MCY, PDB 4XFX, PDB 3H47, PDB 4WYM, PDB 5TSX and PDB 6PU1. Source data are provided with this paper.

## Source data

Source Data Fig. 4 Statistical Source Data
Source Data Extended Data Fig. 1 Statistical Source Data
Source Data Extended Data Fig. 2 Statistical Source Data
Source Data Extended Data Fig. 6 Statistical Source Data
Source Data Extended Data Fig. 7 Statistical Source Data
Source Data Extended Data Fig. 8 Statistical Source Data
