# Supplementary material for: A molecular switch modulates assembly and host factor binding of the HIV-1 capsid
Source: Nat Struct Mol Biol. 2023 Feb 9;30(3):383–90. doi: 10.1038/s41594-022-00913-5 (PMC10023569; doi:10.1038/s41594-022-00913-5)
Supplement: Supplementary file 1 — Reporting Summary [file 41594_2022_913_MOESM1_ESM.pdf]

nature portfolio

Corresponding author(s): Owen Pornillos, Barbie K. Ganser-Pornillos

Last updated by author(s): Dec 5, 2022

Reporting Summary

Nature Portfolio wishes to improve the reproducibility of the work that we publish. This form provides structure for consistency and transparency in reporting. For further information on Nature Portfolio policies, see our Editorial Policies and the Editorial Policy Checklist.

Statistics

For all statistical analyses, confirm that the following items are present in the figure legend, table legend, main text, or Methods section.

n/a	Confirmed	
☐	☒	The exact sample size (*n*) for each experimental group/condition, given as a discrete number and unit of measurement
☒	☐	A statement on whether measurements were taken from distinct samples or whether the same sample was measured repeatedly
☒	☐	The statistical test(s) used AND whether they are one- or two-sided<br>*Only common tests should be described solely by name; describe more complex techniques in the Methods section.*
☒	☐	A description of all covariates tested
☒	☐	A description of any assumptions or corrections, such as tests of normality and adjustment for multiple comparisons
☐	☒	A full description of the statistical parameters including central tendency (e.g. means) or other basic estimates (e.g. regression coefficient) AND variation (e.g. standard deviation) or associated estimates of uncertainty (e.g. confidence intervals)
☒	☐	For null hypothesis testing, the test statistic (e.g. *F*, *t*, *r*) with confidence intervals, effect sizes, degrees of freedom and *P* value noted<br>*Give P values as exact values whenever suitable.*
☒	☐	For Bayesian analysis, information on the choice of priors and Markov chain Monte Carlo settings
☒	☐	For hierarchical and complex designs, identification of the appropriate level for tests and full reporting of outcomes
☒	☐	Estimates of effect sizes (e.g. Cohen's *d*, Pearson's *r*), indicating how they were calculated

Our web collection on statistics for biologists contains articles on many of the points above.

Software and code

Policy information about availability of computer code

Data collection	CryoEM data were collected with EPU 3.
Data analysis	CryoEM data were processed with cryoSPARC v3.3.1-3, utilizing the embedded implementations of MotionCor2 and CTFFIND4 in these versions of cryoSPARC. Coordinate modeling and refinement were done with Coot v0.8.8 and Phenix v1.20.1, utilizing the embedded MolProbity functionalities in Phenix. Structures were also visualized and represented in Chimera v0.8.8 and MacPyMOL v1.8.2.3.

For manuscripts utilizing custom algorithms or software that are central to the research but not yet described in published literature, software must be made available to editors and reviewers. We strongly encourage code deposition in a community repository (e.g. GitHub). See the Nature Portfolio guidelines for submitting code & software for further information.

Data

Policy information about availability of data

All manuscripts must include a data availability statement. This statement should provide the following information, where applicable:

- Accession codes, unique identifiers, or web links for publicly available datasets
- A description of any restrictions on data availability
- For clinical datasets or third party data, please ensure that the statement adheres to our policy

CryoEM maps are deposited at the Electron Microscopy Data Bank (EMDB) under accession numbers EMD-26715 (WT declination), EMD-28054 (T=1 G60A/G61P), EMD-28057 (T=1 M66A), EMD-26718 (T=1 G60A/G61P/M66A) and EMD-28186 (WT declination bound to CPSF6-FG peptide). Coordinates are deposited at the

Protein Data Bank (PDB) under accession numbers 7urn (WT declination), 8ejl (WT-FG complex), 8eep (G61A/G60P), 8eet (M66A) and 7urt (G60A/G61P/M66A). Source data are provided for graphs reported in the paper. The following datasets were used in this study: EMD-3465, EMD-3466, PDB 5mcy, PDB 4xfx, PDB 3h47, PDB 4wym, PDB 5tsx and PDB 6pu1.

Human research participants

Policy information about studies involving human research participants and Sex and Gender in Research.

Reporting on sex and gender	No human research participants
Population characteristics	No human research participants
Recruitment	No human research participants
Ethics oversight	No human research participants

Note that full information on the approval of the study protocol must also be provided in the manuscript.

Field-specific reporting

Please select the one below that is the best fit for your research. If you are not sure, read the appropriate sections before making your selection.

☒ Life sciences ☐ Behavioural & social sciences ☐ Ecological, evolutionary & environmental sciences

For a reference copy of the document with all sections, see nature.com/documents/nr-reporting-summary-flat.pdf

Life sciences study design

All studies must disclose on these points even when the disclosure is negative.

Sample size	For cryoEM structure determinations, sufficient numbers of images were collected with the aim of obtaining at least 100,000 particles in each of the final reconstructions. The final number of particles used for structure calculations are: WT declination, 525,219; WT declination in complex with CPSF6 peptide, 166,463; G60A/G61P, 494,057; M66A, 116,627; G60A/G61P/M66A, 509,666. For data presented in Extended Data Figure 1l, sufficient numbers of images were collected (over n=2 to 10 independent samples) with the aim of obtaining at least 100 particles of each type in the aggregate; all particles were counted, subject to exclusions below. No statistical methods were used to pre-determine sample sizes, following the standard practice in these fields.
Data exclusions	Individual cryoEM images were discarded due to strong drift, devitrification or ice contamination after manual inspection. For data presented in Extended Data Figure 1l, particles were excluded from measurements if their ends (i.e., lengths) cannot be unambiguously determined from the micrograph due to overlap. For data reported in Figure 4e and Extended Data Figure 7, no data were excluded.
Replication	Assembly data in Figure 3b-e and Extended Data Figure 1 were from n=2 to 10 biological replicates; these were collated and analyzed in the aggregate from experiments independently performed by RTS, NFBdS and BKGP. Data presented in Figure 4e and Extended Data Figure 7 are from two biological replicates (3 or 4 technical replicates); one performed by OP and another by NFBdS. All replications (technical and biological) were in very close agreement with each other.
Randomization	For calculation of Fourier shell correlations in cryoSPARC, particle sets were randomly split into two halves and processed independently. Other aspects of the study does not concern experimental determination of co-variances or groupings, and thus randomization is not relevant.
Blinding	Blinding in cryoEM structure determinations is not practiced in the field. For data reported in Extended Data Figure 1l, the experimenters performing the measurements (OP and NFBdS) were unaware of the identities of the samples. However, we note that sample identities can be inferred from the images as they are analyzed.

Reporting for specific materials, systems and methods

We require information from authors about some types of materials, experimental systems and methods used in many studies. Here, indicate whether each material, system or method listed is relevant to your study. If you are not sure if a list item applies to your research, read the appropriate section before selecting a response.

Materials & experimental systems

n/a	Involved in the study
☒	☐ Antibodies
☒	☐ Eukaryotic cell lines
☒	☐ Palaeontology and archaeology
☒	☐ Animals and other organisms
☒	☐ Clinical data
☒	☐ Dual use research of concern

Methods

n/a	Involved in the study
☒	☐ ChIP-seq
☒	☐ Flow cytometry
☒	☐ MRI-based neuroimaging
